# A Simple, Effective, Green Method for the Regioselective 3-Acylation of Unprotected Indoles

**DOI:** 10.3390/molecules201019605

**Published:** 2015-10-27

**Authors:** Phuong Hoang Tran, Hai Ngoc Tran, Poul Erik Hansen, Mai Hoang Ngoc Do, Thach Ngoc Le

**Affiliations:** 1 Department of Organic Chemistry, Faculty of Chemistry, University of Science, Vietnam National University, Ho Chi Minh City 70000, Vietnam; E-Mails: thphuong15@yahoo.com (P.H.T.); sidoantruong@gmail.com (H.N.T.); dhnmai@gmail.com (M.H.N.D.); lenthach@yahoo.com (T.N.L.); 2 Department of Science, Systems and Models, Roskilde University, POB 260, Roskilde DK-4000, Denmark

**Keywords:** Friedel-Crafts acylation, indole derivatives, ionic liquids, metal triflate, microwave irradiation

## Abstract

A fast and green method is developed for regioselective acylation of indoles in the 3-position without the need for protection of the NH position. The method is based on Friedel-Crafts acylation using acid anhydrides. The method has been optimized, and Y(OTf)_3_ in catalytic amounts is found to be the best catalyst together with the commercially available ionic liquid [BMI]BF_4_ (1-butyl-3-methylimidazolium tetrafluoro-borate) as solvent. The reaction is completed in a very short time using monomode microwave irradiation. The catalyst can be reused up to four times without significant loss of activity. A range of substituted indoles are investigated as substrates, and thirteen new compounds have been synthesized.

## 1. Introduction

3-Acylindoles are useful intermediates in the synthesis of various pharmaceuticals [1,2,3,4]. Regioselectivity in the 3-acylation of indoles has been an interesting and challenging subject in organic synthesis. A wide range of 3-acylindoles was synthesized by several methods such as Friedel-Crafts acylation [5,6,7,8,9,10,11,12], Vilsmeier-Haack type reaction [4,13], α-aminocarbonyl compounds with palladium [14], carbamoyl electrophiles [2], α-oxocarboxylic acids [15,16,17], and nitrilium salt with palladium [18]. Among those, Friedel-Crafts acylation of free (NH) indoles is definitely the simplest way [19], however, low yields were observed due to competing substitution at the 1-position. Therefore, *N*-acylated and 1,3-diacylated products were obtained, or indole polymerization may occur in the Friedel-Crafts acylation. To reduce these side products, NH-protection was necessary [8,20]. The protection-deprotection steps are not green and convenient methods [21]. Besides, traditional Lewis acid-catalyzed Friedel-Crafts acylation must be carried out under strictly anhydrous conditions and requires a greater than stoichiometric amount of Lewis acid [22]. Aluminum trichloride, a well-known Lewis acid commonly used in Friedel-Crafts acylations requires a complicated work-up process and causes environmental problems [22]. The development of alternative Lewis acid-catalyzed 3-acylations of indoles has been studied intensively [19]. Among these alternative Lewis acid catalysts, metal triflates were a good option in various organic reactions [19]. In comparison to traditional Lewis acids, metal triflate-catalysed Friedel-Crafts acylation does not require strictly anhydrous conditions due to their water-tolerant characteristics [23]. Besides, only 1–5 mole % of metal triflate is sufficient for complete conversion [23]. Furthermore, this can easily be recovered after workup.

Although metal triflates have been applied extensively in Friedel-Crafts acylation of aromatic compounds [24,25,26,27,28], there has been only one report on the use of indium triflate for the acylation of indoles [29]. However, in that case, excess reagent and NH-protection were required to obtain 3-acylated indoles in good yields. Recently, metal triflates dissolved in ionic liquids were found to be a good catalytic system in Friedel-Crafts acylations [30]. The Friedel-Crafts acylation using metal triflate in ionic liquids has been shown to increase the yield with high regioselectivity and to simplify the recovery of the catalyst [31,32,33,34,35,36,37].

Microwave-mediated organic synthesis provides a useful method due to specific interactions and energy efficiency. Microwave irradiation has allowed the design of efficient processes with significant improvements of yield and selectivity in a short reaction time and simplification of product purification [38].

We report here the development of a new method for the Friedel-Crafts 3-acylation of indoles with various acid anhydrides using metal triflates in ionic liquids under microwave activation. In this paper, we are especially interested in rare-earth metal triflates due to their high catalytic activity and imidazolium ionic liquids because they are commercially available.

## 2. Results and Discussion

The first step was to find the best catalyst from among fourteen metal triflates, including ten rare-earth metal triflates and four well-known triflates such as bismuth triflate, indium triflate, copper triflate and yttrium triflate. The acylation of indole with propionic anhydride was chosen as the model reaction. Indole was treated with 1 mol % of metal triflate in the presence of 1 equiv. of propionic anhydride under microwave irradiation at 120 °C for 5 min. The results are presented in Table 1. Among these triflates, yttrium triflate showed the best catalytic activity for Friedel-Crafts 3-propionylation of indole. Rare-earth metal triflates were also efficient (73%–81%), while bismuth and praseodynium triflates are demonstrated to be less reactive than the others. Interestingly, direct Friedel-Crafts 3-propionylation of indole gave the desired 3-propionylindole without formation of dipropionylated product and polymers, and less than 5% of *N*-propionylated product was found in all cases (with the exception of bismuth triflate, with 9% *N*-propionylindole). Yttrium triflate showed the highest yield and exhibited stronger catalytic activity than the other metal triflates. This metal triflate has been studied extensively in organic synthesis in general [39,40,41,42,43,44,45,46,47,48].

**Table 1:** Effect of metal triflates on Friedel-Crafts propionylation of indoles under microwave irradiation. 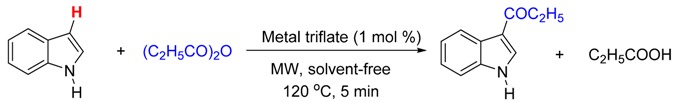

0	Metal Triflate	Isolated Yield (%)	Selective Position (1-/2-/3-)
1	Cu(OTf)_2_	74	5/0/95
2	Y(OTf)_3_	84	3/0/97
3	In(OTf)_3_	80	4/0/96
4	Bi(OTf)_3_	70	9/0/91
5	La(OTf)_3_	81	4/0/96
6	Ce(OTf)_3_	79	4/0/96
7	Pr(OTf)_3_	70	4/0/96
8	Nd(OTf)_3_	74	5/0/95
9	Eu(OTf)_3_	73	4/0/96
10	Gd(OTf)_3_	76	5/0/95
11	Tb(OTf)_3_	76	4/0/96
12	Dy(OTf)_3_	78	4/0/96
13	Ho(OTf)_3_	75	4/0/96
14	Tm(OTf)_3_	80	4/0/96

Next, we tested the effect of solvents on Friedel-Crafts propionylation of indole. The aim of this test was to find the solvent leading to the highest yield with maximum formation of only 3-propionylindole. Eighteen solvents, including traditional organic solvents and commercially available ionic liquids, were investigated. The results are listed in Table 2. The ionic liquid [BMI]BF_4_ was found to be the most effective for Friedel-Crafts propionylation of indole in excellent yield with high regioselectivity, and in this case *N*-propionylation was completely absent. The result indicates that the presence of [BMI]BF_4_ enhances the catalytic activity of yttrium triflate. Friedel-Crafts acylation using metal triflates in ionic liquids has recently attracted attention [31,32,33,34,35,36,37,49]. However, this is the first time that tis catalytic system was applied in the Friedel-Crafts acylation of indoles.

**Table 2:** Effect of solvents under microwave irradiation (80 °C, 5 min) ^a^. 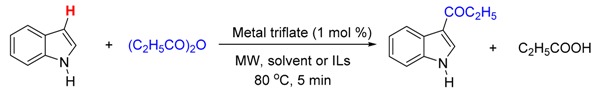

Entry	Solvent	Yield ^b^ (%)	Selective Position (1-/2-/3-) ^c^
1	*n*-hexane	20	5/0/95
2	cyclopentyl methyl ether	50	9/0/91
3	*tert*-butyl methyl ether	27	0/0/100
4	dioxane	63	3/0/97
5	tetrahydrofuran	50	4/0/96
6	ethyl acetate	63	3/0/97
7	chloroform	34	10/0/90
8	dichloromethane	22	0/0/100
9	acetone	44	37/0/63
10	acetonitrile	68	2/0/100
11	methanol	0	-
12	dimethyl carbonate	57	3/0/97
13	[BMI]BF_4_	92	0/0/100
14	[BMI]Cl	66	5/0/95
15	[BMI]PF_6_	70	3/0/97
16	[BMI]SCN	43	22/0/78
17	[BPy]OTf	68	6/0/94
18	[EMI]Cl	74	4/0/96

^a^ Indole (1 mmol), propionic acid anhydride (1 mmol), yttrium triflate (0.01 mmol). ^b^ Isolated yield. ^c^ Isomers were determined by GC.

With optimized conditions in hand, we next investigated the substrate and reagent scope for the Friedel-Crafts 3-acylation of indoles with acid anhydrides using yttrium triflate in [BMI]BF_4_, and the results are presented in Table 3. Indole afforded 3-acylindole in excellent yields with high regioselectivity for the 3-position when using aliphatic acid anhydrides as acylating reagents (Table 3, entries 1–5). Benzoylation of indole gave the desired product in only 78% yield due to competing *N*-benzoylation (Table 3, entry 6). For indoles with electron-rich substituents on the phenyl ring, indoles such as 5-methylindole and 5-methoxyindole, gave 3-acylindoles in yields of 78%–83% without polymerization or side products. The slightly lower yields of 3-acylated products are due to increased substitution at the 1- and 2-position (Table 3, entries 7–18). However, acylation of 4- or 5-haloindoles with aliphatic acid anhydrides produced 3-acylindoles in good yields (Table 3, entries 19–34). In addition, *N*-acylated byproducts were slightly decreased in comparison with electron-rich indoles, but 2-acylindoles were obtained in small amounts (Table 3, entries 19–29). A variety of aliphatic acid anhydrides have been tested. Good yields were obtained for both straight-chain and branched-chain acid anhydrides. Effects due to steric hindrance do not play a role as pivalic acid anhydride is also reactive in general (Table 3). The use of microwave irradiation is probably very important in the case of pivalic acid anhydride as pivaloyl chloride under Friedel-Crafts conditions are known to lead to decarbonylation [50]. The reaction has also been tested using conventional heating for 5 min. at 80 °C with catalyst and in IL. With indole and propionic acid anhydride the yield was 16% (6/0/94) and with butyric acid anhydride 28% (5/0/95). The regioselectivity was worse and much longer times are needed. Looking at regioselectivity in general, it is seen from Table 1 that the catalyst has little influence. From Table 2, most solvents give good regioselectivity. The exceptions are acetone and [BMI]SCN. It is hard to find a common factor for those conditions. It can be added that using propionic anhydride with indole gave a ratio of 7/93 but only in 20% yield in the absence of catalyst. From Table 3, it is seen that pivalic acid anhydride and 2-methylpropionic acid anhydride give rise to more 2-substitution than straight chain acid anhydrides and so does benzoic acid anhydride. A comparison of entries 23 and 34 shows that the reason is not purely steric. More important seems to be the nature of the substitutent in the 5-position. As the three mentioned acylium ions are more stable than those of straight chain acylium ions, it appears that a higher yield of the 2-isomer is caused by a combination of a small steric effect, electronic influence of the 5-position and the stability of the acylium ion.

**Table 3:** Friedel-Crafts acylation of indoles using Y(OTf)_3_/[BMI]BF_4_ under monomode microwave irradiation ^a^. 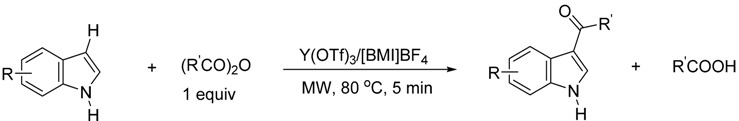

Entry	Substrate	R′	Selective Position in Parenthesis ^b^(1-/2-/3-)	Isolated Yield (%) of 3-Substituted Derivative *^c^*
1	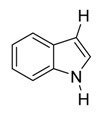	CH_3_	0/0/100	88 ^d^
2		C_2_H_5_	0/0/100	92
3		C_3_H_7_	3/0/97	88 ^e^
4		*i*-C_3_H_7_	2/2/96	91
5		*t*-C_4_H_9_	1/4/95	89
6		C_6_H_5_	5/3/92	78 ^f^
7	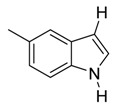	CH_3_	6/0/94	79 ^g^
8		C_2_H_5_	6/1/93	80
9		C_3_H_7_	7/2/91	79
10		*i*-C_3_H_7_	1/8/91	80
11		*t*-C_4_H_9_	1/6/93	83
12		C_6_H_5_	3/4/93	83
13	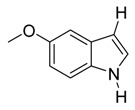	CH_3_	8/0/92	79 ^g^
14		C_2_H_5_	6/2/92	78
15		C_3_H_7_	6/2/92	80
16		*i*-C_3_H_7_	2/7/91	78
17		*t*-C_4_H_9_	1/4/95	79
18		C_6_H_5_	7/4/89	78
19	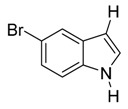	CH_3_	4/2/94	83 ^h^
20		C_2_H_5_	4/1/95	85 ^g^
21		C_3_H_7_	5/2/93	78
22		*i*-C_3_H_7_	2/5/93	80
23		*t*-C_4_H_9_	1/4/95	80 ^g^
24		C_6_H_5_	not determined ^i^	75
25	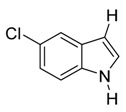	CH_3_	3/1/96	79 ^h^
26		C_2_H_5_	2/2/96	82
27		C_3_H_7_	3/2/95	80
27		*i*-C_3_H_7_	2/7/91	79
29		*t*-C_4_H_9_	6/15/79	65 ^g^
30		C_6_H_5_	1/9/90	77
31	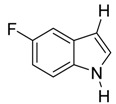	C_2_H_5_	3/3/94	80
32		*t*-C_4_H_9_	3/39/58	45
33	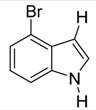	C_2_H_5_	10/0/90	72
34		*t*-C_4_H_9_	4/4/92	82

^a^ Reaction conditions: indoles (1 mmol), acid anhydrides (1 mmol) and Y(OTf)_3_ (0.01 mmol), [BMI]BF_4_ (1 mmol) at 80 °C for 5 min unless otherwise noted. Other conditions were tested to obtain the best yield. ^b^ Selectivity was determined by GC. ^c^ Isolated yield of pure isomer. ^d^ 110 °C, 10 min. ^e^ 100 °C, 5 min. ^f^ 100 °C, 1 min. ^g^ 80 °C, 10 min. ^h^ 100 °C, 10 min, ^i^ the compounds is not suitable for GC analysis.

**Scheme 1:**
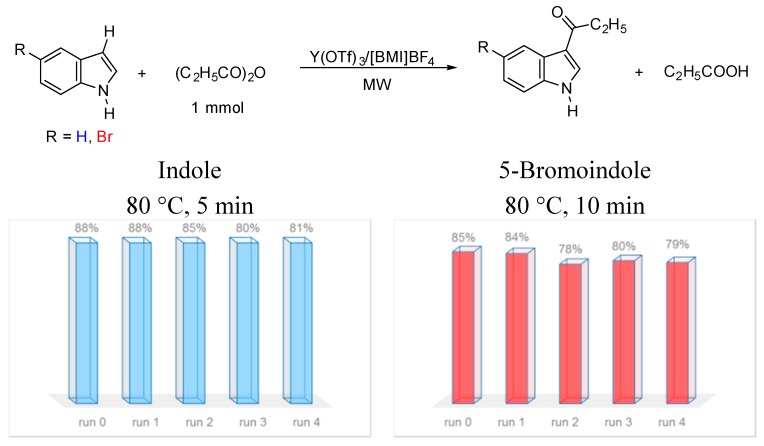
Recycling of Y(OTf)_3_/[BMI]BF_4_ in four consecutive runs under microwave irradiation.

The reusability of Y(OTf)_3_/[BMI]BF_4_ was also studied. After the first use, the recovered catalytic system was tested in four consecutive runs without significant loss of catalytic activity in propionylation of indole and 5-bromoindole (Scheme 1). The recovery of yttrium triflate in [BMI]BF_4_ is simple. After workup, the ionic liquid containing metal triflate is dried under vacuum before the next use.

## 3. Experimental Section 

### 3.1. Chemicals and Supplies

Indoles, acid anhydrides and metal triflates were purchased from Sigma-Aldrich (St. Louis, MO, USA) and immediately used without further purification. Solvents were obtained from Labscan (Bangkok, Thailand) and Chemsol (Hochiminhcity, Vietnam) and also directly used without purification. Silica gel was from Merck (Darmstadt, Germany). 

### 3.2. Instruments

Microwave irradiation was performed in a CEM Discover BenchMate apparatus (Matthews, NC, USA) which allows microwave synthesis with safe pressure regulation using a 10 mL pressurized glass tube with Teflon-coated septum and vertically-focused IR temperature sensor controlling reaction temperature. Flash column chromatography was performed on silica gel (Merck). GC-MS analyses were performed on an Agilent GC System 7890 (Santa Clara, CA, USA) equipped with an Agilent 5973N mass selective detector and a DB-5MS capillary column (30 m × 250 µm × 0.25 µm). The ^1^H- and ^13^C-NMR spectra were recorded on an Advance 500 (Bruker, Rheinstetten, Germany) and Mercury 300 (Varian, Palo Alto, CA, USA) instrument using DMSO-*d*_6_ or CDCl_3_ as solvent and solvent peaks or TMS as internal standards. HRMS (ESI) data were recorded on Bruker micrOTOF-QII MS (Bruker, Bremen, Germany) at 80 eV.

### 3.3. Acylation Procedure 

A 10 mL glass vessel suited for the monomode microwave oven was charged with 1 mmol substrate, 1 mmol acid anhydride, 0.01 mmol metal triflate, and 1 mmol ionic liquid. Next, the vessel was sealed with a Teflon cap and irradiated in a monomode microwave oven at many different reaction conditions (temperature and time) to find the optimal condition. Upon completion, the vessel was cooled down to room temperature and the mixture was extracted with Et_2_O (5 × 10 mL). The ether layer was decanted and washed with water (2 × 10 mL), saturated aqueous NaHCO_3_ (2 × 20 mL), and brine (2 × 10 mL). The organic layer was dried over MgSO_4_, filtered, and the solvent was removed by a rotary evaporator. The isolated yield was determined after purification by flash chromatography (silica gel, *n*-hexane/ethyl acetate, gradient 10:0 to 8:2).

### 3.4. Recovery and Reuse of the Catalytic System Y(OTf)_3_/[BMI]BF_4_

This procedure was also carried out in the monomode microwave oven using indole or 5-bromoindole. In order to recover the catalytic Y(OTf)_3_/[BMI]BF_4_ system after completion of the reaction, diethyl ether was applied to wash the reaction mixture as many times as necessary to completely remove both substrates and products. Then, the mixture Y(OTf)_3_/[BMI]BF_4_ was dried in vacuum at 80 °C for 30 min. Due to its high solubility in the ionic liquid [BMI]BF_4_, Y(OTf)_3_ could easily be recovered in a quantitative yield. This recycled system was used for four consecutive runs and it can be noticed that the isolated yield of product only decreased slightly after each run. The process for recycling Y(OTf)_3_ in [BMI]BF_4_ is simple and efficient so it easily can be applied on a large scale. 

### 3.5. Compounds

New compounds have been synthesized as follows:

*3-Propionyl-5-Methylindole*

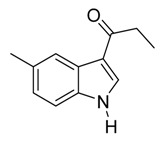

Pale yellow solid, mp. 208–209 °C. ^1^H-NMR (300 MHz, DMSO-*d*_6_): δ 11.75 (br s, 1H), 8.23 (s, 1H), 8.04–7.93 (m, 1H), 7.33 (d, *J* = 8.3 Hz, 1H), 7.02 (dd, *J* = 8.3, 1.5 Hz, 1H), 2.84 (q, *J* = 7.4 Hz, 2H), 2.39 (s, 3H), 1.10 (t, *J* = 7.4 Hz, 3H). ^13^C-NMR (75 MHz, DMSO-*d*_6_): δ 195.7, 134.9, 133.4, 130.3, 125.7, 124.1, 121.0, 115.6, 111.6, 31.8, 21.3, 9.2. GC-MS (EI, 70 eV): *m*/*z* (%) = 187 (25, [M^+^]). HR-ESI-MS *m*/*z* calcd. for ([M + Na]^+^) 210.0889, found 210.0917.

*3-Butyryl-5-methylindole*

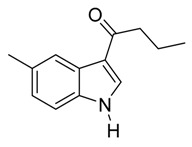

Pale yellow solid, mp. 190–191 °C. ^1^H-NMR (300 MHz, DMSO-*d*_6_) δ 11.75 (br s, 1H), 8.26–8.22 (m, 1H), 8.00 (d, *J* = 2.0 Hz, 1H), 7.30 (s, 1H), 7.01 (s, 1H), 2.77 (t, *J* = 7.3 Hz, 2H), 2.37 (s, 3H), 1.64 (sext, *J* = 7.4 Hz, 2H), 0.91 (t, *J* = 7.4 Hz, 3H). ^13^C-NMR (75 MHz, DMSO-*d*_6_) δ 195.2, 134.9, 133.6, 130.2, 125.6, 124.0, 121.1, 116.1, 111.6, 35.7, 18.3, 13.8. GC-MS (EI, 70 eV): *m*/*z* (%) = 201 (25, [M^+^]). HR-ESI-MS: *m*/*z* calcd. for ([M + Na]^+^) 224.1046, found 224.1053.

*3-Isobutyryl-5-methylindole*

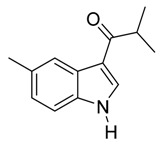

Pale yellow solid, mp. 210–211 °C. ^1^H-NMR (300 MHz, DMSO-*d*_6_): δ 11.76 (br s, 1H), 8.26 (d, *J* = 3.1 Hz, 1H), 8.00 (s, 1H), 7.31 (d, *J* = 8.3 Hz, 1H), 7.00 (dd, *J* = 8.3, 1.6 Hz, 1H), 3.41 (hept, *J* = 6.8 Hz, 1H), 2.37 (s, 3H), 1.09 (d, *J* = 6.8 Hz, 6H). ^13^C-NMR (75 MHz, DMSO-*d*_6_): δ 199.3, 135.0, 133.4, 130.3, 126.0, 124.1, 121.2, 114.6, 111.6, 35.7, 21.3, 19.8. GC-MS (EI, 70 eV): *m*/*z* (%) = 201 (25, [M^+^]). HR-ESI-MS: *m*/*z* calcd. for ([M + Na]^+^) 224.1046, found 224.1063.

*3-Propionyl-5-methoxyindole*

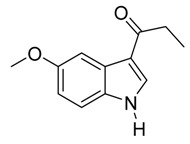

White solid, mp. 182–183 °C. ^1^H-NMR (300 MHz, DMSO-*d*_6_): δ 11.74 (br s, 1H), 8.21 (s, 1H), 7.69 (d, *J* = 2.5 Hz, 1H), 7.33 (d, *J* = 8.8 Hz, 1H), 6.81 (dd, *J* = 8.8, 2.6 Hz, 1H), 3.75 (s, 3H), 2.82 (q, *J* = 7.4 Hz, 2H), 1.09 (t, *J* = 7.4 Hz, 3H). ^13^C-NMR (75 MHz, DMSO-*d*_6_): δ 195.7, 155.3, 133.6, 131.4, 126.1, 115.8, 112.7, 112. 103.0, 55.2, 31.7, 9.1. GC–MS (EI, 70 eV): *m*/*z* (%) = 203 (25, [M^+^]). HR-ESI-MS: *m*/*z* calcd. for ([M + Na]^+^) 226.0839, found 226.0856.

*3-Butyryl-5-methoxyindole*

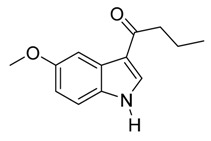

White solid, mp. 166–167 °C. ^1^H-NMR (300 MHz, DMSO-*d*_6_): δ 11.77 (s, 1H), 8.24 (s, 1H), 7.72 (d, *J* = 2.5 Hz, 1H), 7.34 (d, *J* = 8.8 Hz, 1H), 6.83 (dd, *J* = 8.8, 2.6 Hz, 1H), 3.77 (s, 3H), 2.79 (t, *J* = 7.4 Hz, 2H), 1.66 (sext, *J* = 7.4 Hz, 2H), 0.94 (t, *J* = 7.4 Hz, 3H). ^13^C-NMR (75 MHz, DMSO-*d*_6_): δ 195.2, 155.3, 133.8, 131.4, 126.1, 116.3, 112.7, 112.6, 103.0, 55.2, 40.5, 18.3, 13.9. GC–MS (EI, 70 eV): *m*/*z* (%) = 217 (25, [M^+^]). HR-ESI-MS: *m*/*z* calcd. for ([M + Na]^+^) 240.0995, found 240.1026.

*3-Isobutyryl-5-methoxyindole*

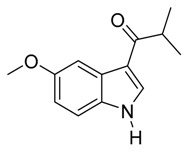

White solid, mp. 158–159 °C. ^1^H-NMR (500 MHz, CDCl_3_): δ 8.77 (br s, 1H), 7.97 (d, *J* = 2.5 Hz, 1H), 7.85 (d, *J* = 3.0 Hz, 1H), 7.29 (d, *J* = 8.8 Hz, 1H), 6.92 (dd, *J* = 8.8, 2.5 Hz, 1H), 3.87 (s, 3H), 3.33 (hept, *J* = 6.8 Hz, 1H), 1.27 (d, *J* = 6.8 Hz, 7H). ^13^C-NMR (125 MHz, CDCl_3_): δ 201.1, 156.4, 131.3, 131.0, 126.7, 116.6, 114.4, 112.1, 103.6, 55.7, 37.1, 19.8. GC-MS (EI, 70 eV): *m*/*z* (%) = 217 (25, [M^+^]). HR-ESI-MS: *m*/*z* calcd. for ([M + Na]^+^) 240.0995, found 240.1035.

*3-Pivaloyl-5-methoxyindole*

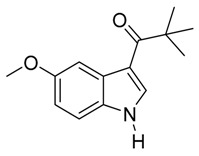

White solid, mp. 153–154 °C. ^1^H-NMR (300 MHz, DMSO-*d*_6_): δ 11.74 (s, 1H), 8.29 (d, *J* = 1.9 Hz, 1H), 7.84 (d, *J* = 2.5 Hz, 1H), 7.33 (d, *J* = 8.8 Hz, 1H), 6.81 (dd, *J* = 8.8, 2.6 Hz, 1H), 3.77 (s, 3H), 1.33 (s, 9H). ^13^C-NMR (75 MHz, DMSO-*d*_6_): δ 201.5, 155.7, 133.1, 131.0, 128.4, 113.0, 112.8, 112.5, 104.1, 55.6, 43.8, 29.1. GC-MS (EI, 70 eV): *m*/*z* (%) = 231 (15, [M^+^]). HR-ESI-MS: *m*/*z* calcd. for ([M + Na]^+^) 254.1152, found 254.1189.

*3-Isobutyryl-5-bromoindole*

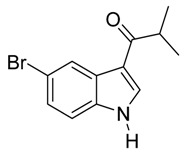

White solid, mp. 223–224 °C. ^1^H-NMR (300 MHz, DMSO-*d*_6_): δ 12.11 (br s, 1H), 8.42 (s, 1H), 8.35 (dd, *J* = 2.0, 0.5 Hz, 1H), 7.44 (dd, *J* = 8.6, 0.5 Hz, 1H), 7.33 (dd, *J* = 8.6, 2.0 Hz, 1H), 3.44 (hept, *J* = 6.8 Hz, 1H), 1.12 (d, *J* = 6.8 Hz, 6H). ^13^C-NMR (75 MHz, DMSO-*d*_6_): δ 199.5, 135.4, 134.7, 127.5, 125.3, 123.6, 114.4, 114.4, 114.1, 35.8, 19.7. GC-MS (EI, 70 eV): *m*/*z* (%) = 265 (25, [M^+^]). HR-ESI-MS: *m*/*z* calcd. for ([M + Na]^+^) 287.9994, found 288.0006.

*3-Pivaloyl-5-bromoindole*

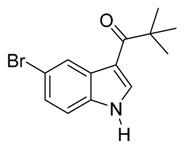

White solid, mp. 234–235 °C. ^1^H-NMR (300 MHz, DMSO-*d*_6_): δ 12.04 (br s, 1H), 8.42 (dd, *J* = 2.0, 0.5 Hz, 1H), 8.40 (s, 1H), 7.41 (dd, *J* = 8.6, 0.5 Hz, 1H), 7.30 (dd, *J* = 8.6, 2.0 Hz, 1H), 1.31 (s, 9H). ^13^C-NMR (75 MHz, DMSO-*d*_6_): δ 201.1, 134.3, 133.6, 128.9, 125.0, 124.1, 114.2, 113.7, 111.6, 43.3, 28.3. GC-MS (EI, 70 eV): *m*/*z* (%) = 279 (15, [M^+^]). HR-ESI-MS: *m*/*z* calcd. for ([M + Na]^+^) 302.0151, found 302.0186.

*3-Butyryl-5-chloroindole*

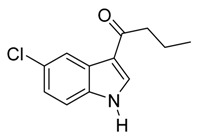

Pale yellow solid, mp. 214–215 °C. ^1^H-NMR (500 MHz, CDCl_3_): δ 8.60 (br s, 1H), 8.42 (d, *J* = 2.0 Hz, 1H), 7.87 (d, *J* = 2.9 Hz, 1H), 7.33 (d, *J* = 8.6 Hz, 1H), 7.24 (*J* = 8.7, 2.1 Hz, 1H), 2.83 (t, *J* = 7.4 Hz, 2H), 1.81 (sext, *J* = 7.4 Hz, 2H), 1.03 (t, *J* = 7.4 Hz, 3H). ^13^C-NMR (125 MHz, CDCl_3_): δ 196.1, 134.6, 131.5, 130.2, 126.6, 124.2, 122.2, 118.1, 112.2, 41.8, 18.4, 14.0. GC-MS (EI, 70 eV): *m*/*z* (%) = 221 (25, [M^+^]). HR-ESI-MS: *m*/*z* calcd. for ([M + Na]^+^) 244.0499, found 244.0472.

*3-Isobutyryl-5-chloroindole*

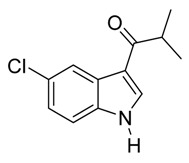

Pale yellow solid, mp. 198–199 °C. ^1^H-NMR (500 MHz, CDCl_3_): δ 8.66 (br s, 1H), 8.44 (d, *J* = 2.0 Hz, 1H), 7.89 (d, *J* = 2.7 Hz, 1H), 7.33 (d, *J* = 8.6 Hz, 1H), 7.24 (dd, *J* = 8.7, 2.4 Hz, 1H), 3.31 (hept, *J* = 6.8 Hz, 1H), 1.26 (d, *J* = 6.8 Hz, 6H). ^13^C-NMR (125 MHz, CDCl_3_): δ 200.4, 134.7, 131.4, 128.6, 127.0, 124.2, 122.3, 116.7, 112.2, 37.3, 19.6. GC-MS (EI, 70 eV): *m*/*z* (%) = 221 (25, [M^+^]). HR-ESI-MS: *m*/*z* calcd. for ([M + Na]^+^) 244.0499, found 244.0537.

*3-Propionyl-4-bromoindole*

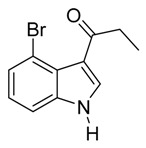

Reddish brown solid, mp. 112–113 °C. ^1^H-NMR (500 MHz, CDCl_3_): δ 9.59 (br s, 1H), 7.40 (dt, *J* = 8.3, 0.8 Hz, 1H), 7.33 (dd, *J* = 7.5, 0.7 Hz, 1H), 7.24 (dd, *J* = 2.3, 0.9 Hz, 1H), 7.19 (dd, *J* = 8.2, 7.6 Hz, 1H), 3.04 (q, *J* = 7.4 Hz, 2H), 1.30 (t, *J* = 7.4 Hz, 3H). ^13^C-NMR (125 MHz, CDCl_3_): δ 194.1, 137.4, 135.0, 128.5, 126.8, 123.8, 116.8, 111.5, 108.9, 31.6, 8.6. GC-MS (EI, 70 eV): *m*/*z* (%) = 251 (70, [M^+^]). HR-ESI-MS: *m*/*z* calcd. for ([M + Na]^+^) 273.9838, found 273.9814.

*3-Pivaloyl-4-bromoindole*

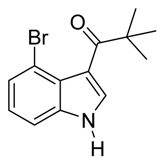

Pale yellow solid, mp = 154 °C. ^1^H-NMR (500 MHz, CDCl_3_): δ 9.37 (br s, 1H), 7.37 (d, *J* = 8.3 Hz, 1H), 7.32 (dd, *J* = 7.5, 0.6 Hz, 1H), 7.25 (dd, *J* = 2.3, 0.9 Hz, 1H), 7.17 (dd, *J* = 8.2, 7.6 Hz, 1H), 1.48 (s, 9H). ^13^C-NMR (125 MHz, CDCl_3_): δ 199.0, 136.1, 132.4, 128.7, 126.6, 123.7, 116.8, 111.2, 108.8, 43.5, 28.4. GC-MS (EI, 70 eV): *m*/*z* (%) = 279 (40, [M^+^]). HR-ESI-MS: *m*/*z* calcd for ([M + Na]^+^) 302.0151, found 302.0189.

## 4. Conclusions

In summary, a new catalytic system Y(OTf)_3_/[BMI]BF_4_ has been developed for 3-acylation of indoles using acid anhydrides under monomode microwave irradiation. This is a simple, straightforward and environmentally benign method to prepare 3-acylindoles in high yields, high regioselectivity and in short reaction times.
